# Prevalence and clinicopathological associations of HER2 expression in non-small cell lung cancer: a retrospective study in Jordanian patients

**DOI:** 10.1186/s13000-023-01364-2

**Published:** 2023-06-20

**Authors:** Ola Abu Al Karsaneh, Arwa Al Anber, Mohammad ALQudah, Sahar Al-Mustafa, Hussien AlMa’aitah, Maher Sughayer

**Affiliations:** 1 Department of Microbiology, Pathology and Forensic Medicine, Faculty of Medicine, The Hashemite University, Zarqa, 13133 Jordan; 2 Department of Pharmacology and Public Health, Faculty of Medicine, The Hashemite University, Zarqa, 13133 Jordan; 3 Department of Pathology and Laboratory Medicine, King Hussein Cancer Center, Amman, Jordan

**Keywords:** Non-small cell lung cancer, Epidermal growth factor receptor, HER2, Survival, Immunohistochemistry, Jordan

## Abstract

**Background:**

Human epidermal growth factor receptor 2 (HER2), a promising therapeutic target, can be mutated, amplified, or overexpressed in different malignancies, including non-small cell lung cancer (NSCLC). Although these alterations showed adverse prognostic effects in many cancers, their clinical significance in NSCLC is controversial. This study primarily assessed the prevalence of HER2 protein expression in NSCLC among Jordanian patients. In addition, the possible association between HER2 protein expression and clinicopathological variables was evaluated.

**Methods:**

A total of 100 surgically resected NSCLC cases treated at King Hussein Cancer Center (KHCC) between 2009 and 2021 were examined for HER2 protein expression using immunohistochemistry (IHC). The American Society of Clinical Oncology/College of American Pathologists (ASCO/CAP) guidelines for breast cancer were applied to interpret the results with a final score ranging from 0 to 3+, considering a score of 3 + as overexpression. Additionally, a separate subset of patients was tested for HER2 gene mutation. Fisher’s exact test was used to assess the association between HER2 scores and the other variables. Kaplan-Meier method was used to calculate survival.

**Results:**

Of the 100 cases, Her2 overexpression (score 3+) was detected in 2 cases (2%), score 2 + in 10 cases (10%), score 1 + in 12 cases (12%), and score 0 in 76 cases (76%). The two positive cases were one adenocarcinoma and one squamous cell carcinoma; both patients were elderly male smokers. No significant association was identified between Her2 expression and age, gender, smoking, histological subtype, grade, stage, tumor size, and lymph node status. Our findings also showed no association between Her2 expression and survival; however, advanced tumor stages and positive lymph node metastasis were significantly associated with poor overall survival. All cases tested for the Her2 mutation were negative.

**Conclusions:**

Her2 overexpression is uncommon in NSCLC among the Jordanian population. However, when the same scoring criteria are used, the rates are similar to other results found in Asian cohorts. Due to our study’s relatively small sample size, a larger one is required to investigate the prognostic value and the molecular associations between the different Her2 alterations.

## Background

Lung cancer is a primary cause of cancer-related deaths for both genders worldwide [1]. Non-small cell lung cancer (NSCLC) accounts for about 85% of lung cancer cases [2]. Although lung cancer is the third most frequently diagnosed cancer in Jordan, it is the most common lethal one [3]. Despite the advancement of lung cancer diagnosis, surgical treatment, and chemotherapy, the overall five-year survival is around 15%, which is one of the lowest among cancers. Several independent prognostic factors have been identified and used to predict the survival of lung cancer patients; among these, different potential biomarkers have emerged and been used to predict an individual outcome.

Lung cancer is driven by different molecular alterations, including the epidermal growth factor receptor (EGFR) mutations and the rearrangements of the anaplastic lymphoma kinase (ALK) and the c-ros1 (ROS1) genes. Targeted therapies are generally recommended for patients carrying these specific genetic alterations [4]. More extensive molecular testing of lung cancer, including next-generation sequencing (NGS), has identified additional potential driver mutations [5]. Among these mutations, the gene encoding human epidermal growth factor receptor 2 (called HER2, EGFR2, NEU, or ERBB2) is a hot new therapeutic target.

HER2 is a glycoprotein encoded by the *HER2* gene, a proto-oncogene found on the long arm of chromosome 17. It is one of the epidermal growth factor receptor (EGFR) family of receptor tyrosine kinases, which additionally includes three different members: EGFR (or HER1/ ERBB1), HER3 (or ERBB3), and HER4 (or ERBB4). Despite sharing structural similarities with the other EGFR family members, HER2 lacks an identified directly activating ligand and instead is activated either through homodimerization or heterodimerization with other members, which activates downstream signaling through the phosphatidylinositide 3-kinase (PI3K)–protein kinase B (AKT) and mitogen-activated protein kinase (MEK) pathways ending with cell proliferation and differentiation [5, 6].

HER2 dysregulation has been linked to the development of numerous human cancers [7]. In breast cancer, studies showed an overall frequency of HER2 overexpression of around 22%, which was found to have an adverse prognostic effect regardless of the lymph node status [8]. In 90% of the cases, HER2 amplification leads to this overexpression [8]. Similarly, HER2 amplification and/ or overexpression have also been detected in subgroups of gastric [9], esophageal [10], and colorectal [11] carcinomas.

According to the ASCO/CAP guidelines, breast cancer is considered HER2-positive when it shows a score of 3 + by IHC or 2 + IHC with gene amplification by in-situ hybridization (ISH). In contrast, those having scores 0 and 1 + by IHC, or 2 + with a negative ISH, are classified as HER2-negative [12]. In recent years, many anti-HER2 drugs have been licensed by the Food and Drug Administration (FDA) to treat individuals with breast cancer that is HER2-positive [13]. Of these, the monoclonal antibody trastuzumab (Herceptin), which targets different extracellular regions of the HER2 receptor, has been successfully used in patients with advanced breast cancer and showed improved survival when combined with cytotoxic chemotherapy [14]. Therefore, measuring HER2 protein overexpression by IHC or gene amplification by fluorescence in situ hybridization (FISH) is applied to select candidate patients for this antibody therapy. Of interest, recently, HER2 “low” breast cancers, those showing 1 + score by IHC or 2 + with negative ISH, have demonstrated some remarkable response rates and progression-free survival after Her2 antibody-drug conjugates (ADC) treatments [15]. Therefore, these findings may change the current Her2 testing and assessment paradigm, which can be expanded to include other cancer types, including lung cancer.

Several studies assessed HER2 oncogene and protein expression in NSCLC, including squamous cell carcinoma, adenocarcinoma, and large cell carcinoma, and three distinct types of HER2 changes have been identified: protein overexpression, gene amplification, and mutations. The reported incidence of HER2 protein overexpression using IHC and gene amplification is 2.4–38% and 10–20%, respectively [16–20]. HER2 mutations in NSCLC were first reported in 2004, with an incidence of up to 4% [21]. Unlike breast cancer, the molecular associations between these alterations were controversial in NSCLC [22, 23].

The effect of HER2 alterations on patients’ prognosis was also contradictory; however, two large meta-analyses showed that HER2 protein overexpression has adverse effects on prognosis in patients with lung cancer [20, 24]. The effects of anti-HER2 therapy in HER2-abnormal NSCLC were also more intricate and differed according to the type of alteration [25–28]. Overall, the uncertain correlations between the various HER2 alterations and the different clinical impacts indicate that their role in lung carcinogenesis is still obscure and that these alterations may represent distinct changes that should be measured separately.

To the best of our knowledge, this is the first study to address the frequency of HER2 protein expression in NSCLC among Jordanian or Middle Eastern/ North African (MENA) patients. Thus, the primary goal of this study was to assess the prevalence of HER2 protein expression among Jordanian patients with NSCLC using IHC. In addition, we assessed any possible associations between the different HER2 protein expression patterns, various clinicopathological variables, and overall patients’ survival.

## Methods

### Patients and tissue samples

**Fig. 1:**
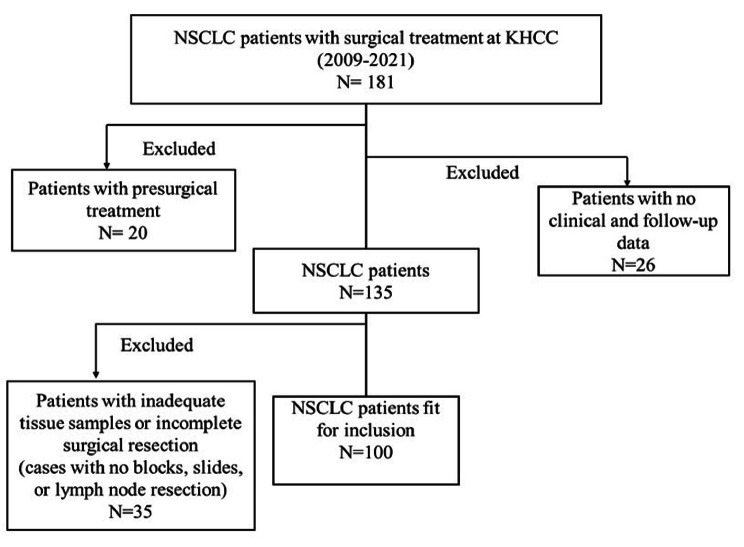
**Flow chart elucidating the inclusion and exclusion criteria of NSCLC patients** The flow chart illustrates that a total of 181 non-small cell lung cancer patients who had surgical treatment as first-line therapy for their cancer at KHCC in 2009–2021 were initially reviewed. Only 100 patients were included in the study after excluding those with presurgical treatment, inadequate clinical and follow-up data, or inadequate tissue samples for evaluation. NSCLC: non-small cell lung cancer. KHCC: King Hussein Cancer Center

A retrospective archival search of the surgically resected NSCLC cases at the department of pathology and laboratory medicine at KHCC, Amman, Jordan, between January 2009 and February 2021 was performed. The patient’s inclusion criteria were surgical treatment of histologically confirmed NSCLC with no preoperative chemotherapy, radiotherapy, or other treatment and having adequate tissue samples and clinical follow-up data. After the exclusion of the patients who had received preoperative treatment, had inadequate tissue samples, or had limited clinical and follow-up data, only 100 patients were fit for inclusion. Figure (1) represents the patient’s inclusion and exclusion criteria flow chart. The archived formalin-fixed, paraffin-embedded (FFPE) blocks and the respective hematoxylin and eosin (H&E) stained sections of the patients were collected. The patients’ relevant clinicopathological data, including age, sex, smoking history, treatment history, histologic subtypes, and pathologic stage, were obtained from the patient’s medical records and pathological reports. To confirm the histologic features, the H&E-stained sections were independently evaluated by two pathologists (O.A.A.K and S.A). Histologic subtypes and tumor grades were determined according to the World Health Organization (WHO) recommendations [29, 30]. Briefly, tumors predominantly having a lepidic architecture with less than 20% of high-grade patterns (solid, micropapillary, cribriform, or complex glands) were classified as well-differentiated adenocarcinoma (ADC); tumors predominantly showing acinar and papillary patterns with less than 20% of high-grade patterns were classified as moderately differentiated ADC, and any ADC with more than 20% of high-grade patterns was categorized as poorly differentiated. Following this, tumor grades were divided into two categories: low-grade, which comprised cancer with well to moderate differentiation, and high-grade, which included those with poor differentiation. The grading of squamous cell carcinoma (SqCC) was determined based on the degree of tumor keratinization. Stages were determined according to the 7th-8th editions of the American Joint Committee (AJC) on the Cancer TNM classification system [31, 32].

### Immunohistochemistry and scoring criteria

The surgically resected specimens were immersed and fixed in 10% neutral- buffered formalin (NBF) within 5 min of the resection and fixed for a duration between 12 and 28 h. Immunohistochemistry was performed using the automated Ventana / ROCHE BenchMark ULTRA IHC/ISH System (Ventana Medical Systems Inc., Tucson, AZ, USA) and following a standard protocol. We used the pre-diluted (ready-to-use) PATHWAY anti-HER2/neu (4B5) rabbit monoclonal primary antibody (Ventana Medical Systems, Tucson, AZ, USA) to detect HER2 protein expression. Briefly, tissue sections of 3 μm thickness were sliced from the FFPE NSCLC specimens, prepared on immunohistochemistry (IHC) adhesive slides (TOMO®), and put in an oven for about 15 min at 65– 70 °C to help the sections adhere to the slides. Deparaffinization was performed using the E.Z. prep solution, which warmed up the slides to 72 °C for 4 min to remove the paraffin wax. Antigen retrieval was done using the Heat-Induced Epitope Retrieval (HIER) in Tris-EDTA buffer pH 7.8 at 95 °C for 36 min (ultra CC1). Blocking was done with Inhibitor CM at 36 °C for 4 min. Then the sections were incubated with the anti-HER2 monoclonal primary antibody for 12 min at 36 °C. Signal detection was carried out with the *ultra*View DAB detection kit by applying the following, one drop of OmniMap anti-Rb H.R.P. and incubation for 16 min, then one drop of DAB CM and One Drop of H2O2 CM with incubation for 8 min, and finally applying one drop of Copper CM and incubation for 4 min. The BenchMark ULTRA instrument washed the sections following each incubation step to prevent the reaction and eliminate the unbound material that would disrupt the desired reaction in the following steps. Counterstaining was performed using Hematoxylin II at 36 °C for 4 min, and after that, post-counterstaining bluing was done at 36 °C for 4 min. After that liquid coverslip was applied to minimize the aqueous reagents’ evaporation from the specimen slides, and when the run finished, the slides were washed in warm water tap with detergent (soap), dehydrated in graded ethanol from 70% to absolute 100% and cleared by xylene. Finally, the slides were coverslipped with dibutyl phthalate in xylene (D.P.X.) mounting media. Positive controls were performed by staining Her2- positive breast cancer samples from the archive of pathology specimen, and surrounding benign lung tissue was utilized as an internal negative control. Two pathologists examined and scored all specimens independently (O.A.A.K and S.A.) using a light microscope (Olympus BX53, Japan), and a consensus was reached on all cases. Cases with scores of 2 + and 3 + were confirmed by a third pathologist (M.S). Scoring followed the guidelines of the ASCO/CAP for breast cancer [33]. Based on membranous staining, the final score varied from 0 to 3+ (0: no staining or incomplete faint staining that comprises < or = 10% of the tumor cells; 1+: incomplete membranous staining that is faint/ weak and constitutes > 10% of the tumor cells; 2+: membrane staining that is incomplete and weak to moderate and constitutes > 10% of the tumor cellularity, or complete membrane staining that is strong but detected in < or = 10% of the tumor cells; and 3+: circumferential staining that is complete and strong in > 10%). Zero and 1 + scores were considered negative, 2 + as equivocal, and 3 + as positive or overexpressed. Since the equivocal (score 2+) cases were not tested for gene amplification by FISH, only cases with 3 + scores were considered positive. Nuclear and cytoplasmic staining alone were considered negative scores.

### Her2 mutation analysis

Her2 mutation status was tested in a separate subset composed of 10 patients who were submitted for FoundationOne CDx (F1CDx) diagnostic test, which employs the next-generation sequencing (NGS)-based comprehensive genomic profiling (CGP) approach to evaluate multiple cancer genes in solid tumors including Her2. The samples were submitted for genetic testing within a few months of the diagnosis. DNA was extracted from routine FFPE biopsy or surgical resection specimens using the 96-well KingFisher™ FLEX Magnetic Particle Processor. Whole genome library construction with the NEBNext® reagents (custom-filled kits by NEB) and hybridization-based capture of DNA was performed. After that, uniform sequencing on the Illumina® HiSeq 4000 platform was done. Following sequencing, custom software was used to determine genomic variants. The targeted median coverage was > 500X, with > 99% of exons at coverage > 100X.

### Statistical analysis

Fisher’s exact test was utilized to assess the relationship between HER2 scores and clinicopathological characteristics. Overall survival (OS) was determined from the time of surgery to the time of death or the last follow-up appointment, and Disease-free survival (DFS) was measured from the time of surgery to that of cancer recurrence/ progression or the last follow-up visit. Using the Kaplan-Meier method, survival rates were computed, and the log-rank test was carried out to analyze the differences. All statistical analyses were conducted on a two-tailed basis, and P-values of ≤ 0.05 were regarded as statistically significant. IBM SPSS statistics software for Windows version 29 (IBM Corp., New York, USA) was utilized for statistical analysis.

## Results

### Patients’ clinicopathological characteristics

The main clinicopathological characteristics of the patients are summarized in Table 1. The cohort that was used for immunohistochemistry was composed of 100 patients who had surgical resection of their NSCLC. Most patients (84%) underwent lobectomy with free margins except for 5 cases which were positive for vascular or bronchial margins involvement by tumor. Eleven patients underwent wedge resection with free margins except for one case with vascular margin involvement, and five patients had pneumonectomy, with one case showing bronchial margin involvement by tumor. Among the whole cohort, 82% were males, 18% were females, and 77% were older than 60 years, with a median age of 68.5 (range 33–81 years). Regarding their smoking habits, 80.6% were either former or current smokers, while 19.4% had never smoked. Adenocarcinoma comprised 75% of our cohort’s histological subtypes, squamous cell carcinoma 24%, and adenosquamous carcinoma morphology only accounted for one case. Most cases were high grades with poor differentiation (62%). The majority of cases were of early stage, and the frequencies of the pathological stages were as follows: stage I (37.1%), stage II (34%), stage III (23.7%), and stage IV (5.2%). Over half of the cases had a tumor size of more than 3 cm (59%), and 63% were negative for lymph node metastasis. Regarding the adenocarcinoma cases, 44% showed an acinar pattern, 29.3% solid pattern, 24% lepidic pattern, and 2.7% micropapillary pattern as predominant architectural patterns. Regarding postoperative treatment, around half of the patients (53%) did not receive any postoperative adjuvant therapy, 32% received adjuvant chemotherapy, 6% received radiation therapy, and 9% received a combination of chemo-radiation therapy. Twenty -nine patients developed disease recurrence either in the form of local recurrence or recurrent metastatic tumor. Of these, eight cases did not receive any further treatment, nine cases received radiation therapy, two cases received chemotherapy, four cases received a combination of chemo-radiation therapy, three cases received a combination of chemotherapy and immunotherapy, one case received a combination of chemotherapy and EGFR tyrosine kinase inhibitor, one case received EGFR tyrosine kinase inhibitor alone, and one case was treated with ALK inhibitor. In addition, driver genes mutation status was available for a limited number of cases as follows; ALK1 was tested in 37 cases where only two cases were positive by IHC. ROS1 was tested by IHC in seven cases, and only one case was positive. EGFR gene was assessed for mutations in 13 cases, and only two cases showed exon 19 deletion and L858R mutation, while other cases showed wild-type EGFR gene. None of the cases carrying a positive driver gene mutation showed Her2 overexpression.

**Table 1:** Clinicopathological characteristics of the patients according to the different Her2 scores

Variables, n (%)	Total	Her2 score			
		Score 0	Score 1+	Score 2+	Score 3+
	100 (100.0)	76 (76.0)	12 (12.0)	10 (10.0)	2 (2.0)
**Age (Years)**					
≤ 60	23 (23.0)	14 (18.4)	4 (33.3)	5 (50.0)	0 (0.0)
> 60	77 (77.0)	62 (81.6)	8 (66.7)	5 (50.0)	2 (100.0)
**Gender**					
Male	82 (82.0)	61 (80.3)	11 (91.7)	8 (80.0)	2 (100.0)
Female	18 (18.0)	15 (19.7)	1 (8.3)	2 (20.0)	0 (0.0)
**Smoking history** *					
Never smoker	19 (19.4)	15 (20.3)	2 (16.7)	2 (20.0)	0 (0.0)
Former/ current smoker	79 (80.6)	59 (79.7)	10 (83.3)	8 (80.0)	2 (100.0)
**Histological subtype**					
Adenocarcinoma	75 (75.0)	55 (72.4)	10 (83.3)	9 (90.0)	1(50.0)
Squamous cell carcinoma	24 (24.0)	20 (26.3)	2 (16.7)	1 (10.0)	1 (50.0)
Adenosquamous carcinoma	1 (1.0)	1 (1.3)	0 (0.0)	0 (0.0)	0 (0.0)
**Differentiation**					
Low-grade (well and moderately differentiated)	38 (38.0)	31(40.8)	3 (25.0)	4 (40.0)	0 (0.0)
High-grade (poorly differentiated)	62 (62.0)	45 (59.2)	9 (75.0)	6 (60.0)	2 (100.0)
**Tumor size**					
≤ 3	41 (41.0)	34 (44.7)	5 (41.7)	2 (20.0)	0 (0.0)
> 3	59 (59.0)	42 (55.3)	7 (58.3)	8 (80.0)	2 (100.0)
**Lymph nodes metastasis**					
Positive	37 (37.0)	26 (34.2)	7 (58.3)	3 (30.0)	1 (50.0)
Negative	63 (63.0)	50 (65.8)	5 (41.7)	7 (70.0)	1 (50.0)
Pathological stage **					
I	36 (37.1)	29 (39.2)	2 (18.2)	4 (40.0)	1(50.0)
II	33 (34.0)	24 (32.4)	5 (45.5)	3 (30.0)	1(50.0)
III	23 (23.7)	18 (24.3)	2 (18.2)	3 (30.0)	0 (0.0)
IV	5 (5.2)	3 (4.1)	2 (18.2)	0 (0.0)	0 (0.0)
**Predominant histological pattern (For ADC cases, 75 cases)**					
Lepidic	18 (24.0)	12 (21.8)	2 (20.0)	4 (44.4)	0 (0.0)
Acinar	33 (44.0)	26 (47.3)	4 (40.0)	2 (22.2)	1 (100.0)
Papillary	0 (0.0)	0 (0.0)	0 (0.0)	0 (0.0)	0 (0.0)
Micropapillary	2 (2.7)	1 (1.8)	0 (0.0)	1 (11.1)	0 (0.0)
Solid	22 (29.3)	16 (29.1)	4 (40.0)	2 (22.2)	0 (0.0)

* Indicates that two patients were excluded due to lack of smoking history, ** indicates that three patients were excluded as the specific stages were not determined. P-values of ≤ 0.05 were regarded as statistically significant. n = number

Regarding the small subset of patients (10 patients) submitted for CGP mutational analysis, 6 were males, and four were females with a mean age of 60.1 (range 25–88 years). Regarding their smoking history, five were current or former smokers; two were nonsmokers, and three lacked data regarding smoking history. Nine cases were adenocarcinoma, and only one case was squamous cell carcinoma. In 90% of the cases, the mutational analysis was performed on a biopsy with no resection performed, and all cases were negative for Her2 mutation.

### HER2 expression and its correlation with clinicopathological characteristics

**Fig. 2:**
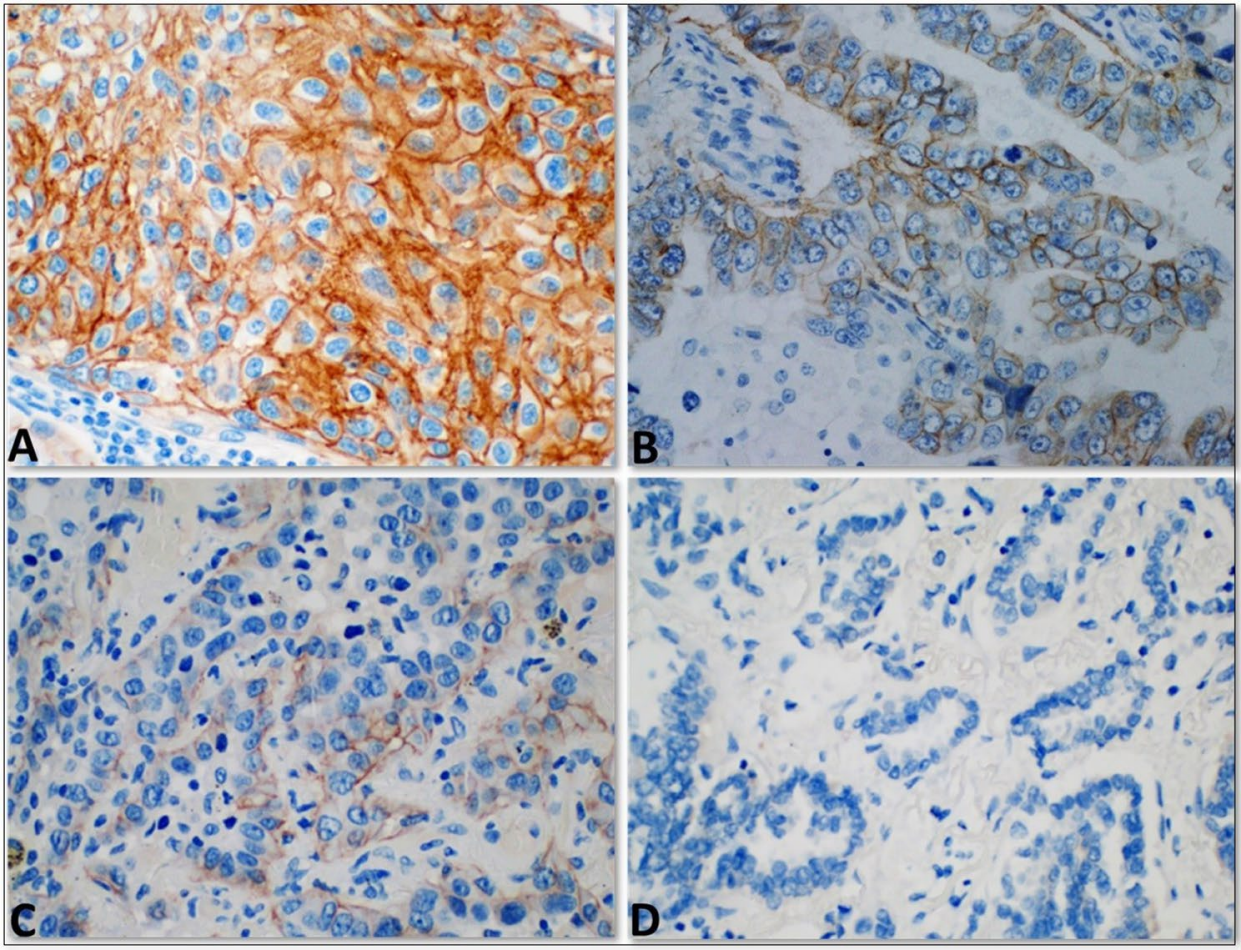
**Representative images of Her2 immunohistochemistry** (**A**) A case of squamous cell carcinoma with complete intense membranous staining of Her2, score 3+; (**B**) A case of adenocarcinoma with incomplete (basolateral) moderate membranous staining of Her2, score 2+; (**C**) A case of adenocarcinoma with incomplete faint/weak membranous staining of Her2, score 1+; (**D**) A case of adenocarcinoma with complete negative staining of Her2, score 0. All images were obtained at 40X magnification

Of the evaluated 100 cases, membranous Her2 expression was scored as 3 + in 2 cases (2%), 2 + in 10 cases (10%), 1 + in 12 cases (12%), and 0 in 76 cases (76%), according to ASCO/CAP guidelines of breast cancer, Fig. (2 A-D), figures were taken using Olympus EP50 camera, Japan. Nuclear and cytoplasmic expression without membranous staining were considered negative. The adjacent normal lung tissue was negative for staining in all cases. The two positive (3+) cases were males, older than 60 years, and had a history of smoking. One case was of adenocarcinoma histology, and the other was squamous cell carcinoma; both were of high- grade but with early stages (stages I and II). The clinicopathological features of the patients according to the different Her2 scores are provided in Table 1. No statistically significant correlation was identified between Her2 expression and the different clinicopathological variables, including age, gender, smoking, grades, and stages; this may be attributed to the very low number of positive cases; these associations are represented in Table 2. Further, we tried to find any correlation between the same clinicopathological variables and Her2 expression by dividing the cases into two groups, one including the completely negative cases (score 0) and the other including those with any level of positivity (scores 1+, 2 + and 3+). However, similarly, no statically significant association was identified, Table 3.

**Table 2:** Clinicopathological correlation with Her2 expression

Variables, n (%)	Total	Her2 Negative or equivocal (scores 0, 1+, and 2+)	Her2 Positive (score 3+)	P value
	100 (100.0)	98 (98.0)	2 (2.0)	
**Age (Years)**				1
≤ 60	23 (23.0)	23 (23.5)	0 (0.0)	
> 60	77 (77.0)	75 (76.5)	2 (100.0)	
**Gender**				1
Male	82 (82.0)	80 (81.6)	2 (100.0)	
Female	18 (18.0)	18 (18.4)	0 (0.0)	
**Smoking history** *				1
Never smoker	19 (19.4)	19 (19.8)	0 (0.0)	
Former/ current smoker	79 (80.6)	77 (80.2)	2 (100.0)	
**Histological subtype**				0.439
Adenocarcinoma	75 (75.0)	74 (75.5)	1(50.0)	
Squamous cell carcinoma	24 (24.0)	23 (23.5)	1 (50.0)	
Adenosquamous carcinoma	1 (1.0)	1 (1)	0 (0.0)	
**Differentiation**				0.524
Low-grade (well and moderately differentiated)	38 (38.0)	38 (38.8)	0 (0.0)	
High-grade (poorly differentiated)	62 (62.0)	60 (61.2)	2 (100.0)	
**Tumor size**				0.511
≤ 3	41 (41.0)	41(41.8)	0 (0.0)	
> 3	59 (59.0)	57 (58.2)	2 (100.0)	
**Lymph nodes metastasis**				1
Positive	37 (37.0)	36 (36.7)	1 (50.0)	
Negative	63 (63.0)	62 (63.3)	1 (50.0)	
Pathological stage **				1
I	36 (37.1)	35 (36.8)	1 (50.0)	
II	33 (34.0)	32 (33.7)	1 (50.0)	
III	23 (23.7)	23 (24.2)	0 (0.0)	
IV	5 (5.2)	5 (5.3)	0 (0.0)	
**Predominant histological pattern (For ADC cases, 75 cases)**				1
Lepidic	18 (24.0)	18 (24.3)	0 (0.0)	
acinar	33 (44.0)	32 (43.2)	1 (100.0)	
Papillary	0 (0.0)	0	0 (0.0)	
Micropapillary	2 (2.7)	2 (2.7)	0 (0.0)	
Solid	22 (29.3)	22 (29.7)	0 (0.0)	

* Indicates that two patients were excluded due to lack of smoking history, ** indicates that three patients were excluded as the specific stages were not determined. P-values of ≤ 0.05 were regarded as statistically significant. n = number

**Table 3:** Clinicopathological correlation with Her2 expression (completely negative and any level of positivity)

Variables, n (%)	Total	Completely Negative Her2 (Score 0)	Any level of Her2 Positivity (3+, 2+, 1+)	P value
	100 (100.0)	76 (76)	24 (24)	
**Age (Years)**				0.092
≤ 60	23 (23.0)	14 (18.4)	9 (37.5)	
> 60	77 (77.0)	62 (81.6)	15 (62.5)	
**Gender**				0.550
Male	82 (82.0)	61 (80.3)	21 (87.5)	
Female	18 (18.0)	15 (19.7)	3 (12.5)	
**Smoking history** *				1
Never smoker	19 (19.4)	15 (20.3)	4 (16.7)	
Former/ current smoker	79 (80.6)	59 (79.7)	20 (83.3)	
**Histological subtype**				0.557
Adenocarcinoma	75 (75.0)	55 (72.4)	20 (83.3)	
Squamous cell carcinoma	24 (24.0)	20 (26.3)	4 (16.7)	
Adenosquamous carcinoma	1 (1.0)	1 (1.3)	0 (0.0)	
**Differentiation**				0.345
Low-grade (well and moderately differentiated)	38 (38.0)	31 (40.8)	7 (29.2)	
High-grade (poorly differentiated)	62 (62.0)	45 (59.2)	17 (70.8)	
**Tumor size**				0.235
≤ 3	41 (41.0)	34 (44.7)	7 (29.2)	
> 3	59 (59.0)	42 (55.3)	17 (70.8)	
**Lymph nodes metastasis**				0.338
Positive	37 (37.0)	26 (34.2)	11 (45.8)	
Negative	63 (63.0)	50 (65.8)	13 (54.2)	
Pathological stage **				0.664
I	36 (37.1)	29 (39.2)	7 (30.4)	
II	33 (34.0)	24 (32.4)	9 (39.1)	
III	23 (23.7)	18 (24.3)	5 (21.7)	
IV	5 (5.2)	3 (4.1)	2 (8.7)	
**Predominant histological pattern (For ADC cases, 75 cases)**				0.585
Lepidic	18 (24.0)	12 (21.8)	6 (30.0)	
acinar	33 (44.0)	26 (47.3)	7 (35.0)	
Papillary	0 (0.0)	0 (0.0)	0 (0.0)	
Micropapillary	2 (2.7)	1 (1.8)	1 (5.0)	
Solid	22 (29.3)	16 (29.1)	6 (30.0)	

* Indicates that two patients were excluded due to lack of smoking history, ** indicates that three patients were excluded as the specific stages were not determined. P-values of ≤ 0.05 were regarded as statistically significant. n = number

### Survival analysis of HER2 expression and other clinicopathological characteristics

**Fig. 3:**
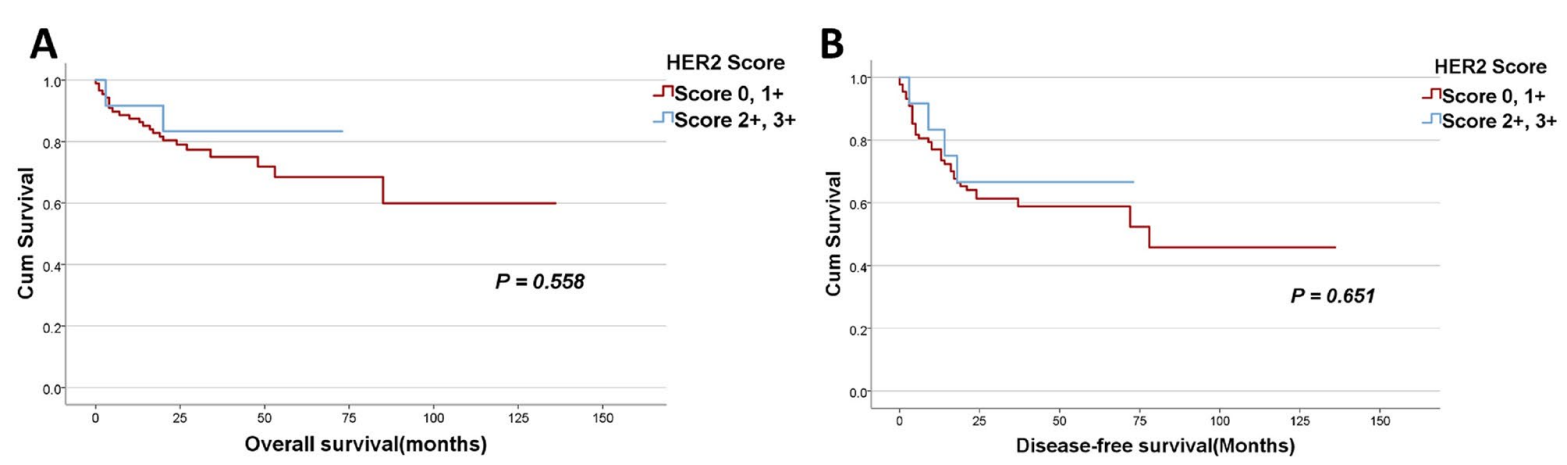
**Kaplan – Meier survival curves according to the different groups of HER2 scores** **A**: The correlation between the different Her2 scores and overall survival (OS) by dividing Her2 scores into two groups: one comprising scores 0 and 1 + and another comprising scores 2 + and 3+. OS was defined as the duration from the time of surgery to the time of death or the last follow-up appointment; **B**: The correlation between the different Her2 scores and disease-free survival (DFS) by dividing Her2 scores into two groups: one comprising scores 0 and 1 + and another one comprising scores 2 + and 3+. DFS was measured from the time of surgery to that of cancer recurrence/ progression or the last follow-up visit. P-values of ≤ 0.05 were regarded as statistically significant

**Fig. 4:**
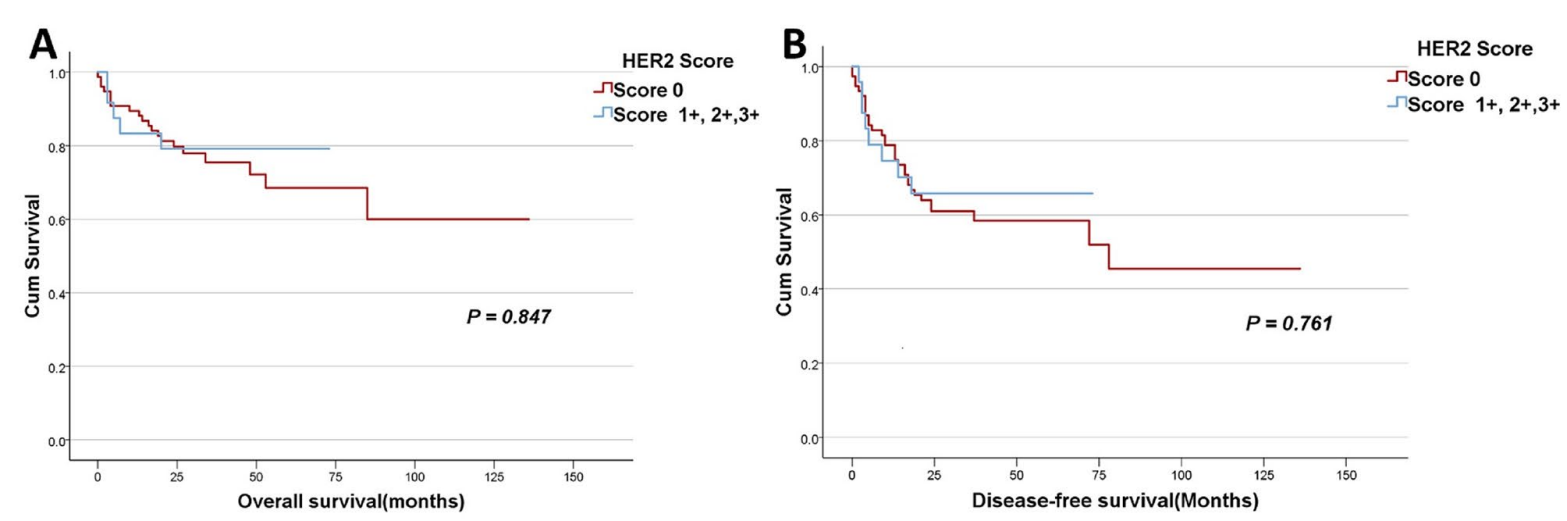
**Kaplan – Meier survival curves according to the different groups of HER2 scores** **A**: The correlation between the different Her2 scores and overall survival (OS) by dividing Her2 scores into two groups: one composed of cases with score 0 and another comprising cases with any level of positivity (scores 1+, 2 + and 3+). OS was defined as the duration from the time of surgery to the time of death or the last follow-up appointment; **B**: The correlation between the different Her2 scores and disease-free survival (DFS) by dividing Her2 scores into two groups: one composed of cases with score 0 and another comprising cases with any level of positivity (scores 1+, 2 + and 3+). DFS was measured from the time of surgery to that of cancer recurrence/progression or the last follow-up visit. P-values of ≤ 0.05 were regarded as statistically significant

The median follow-up period of the patients was 26 months (range 0- 136 months) after surgical resection. No statistically significant difference in overall survival (OS) and disease-free survival (DFS) was found by comparing the patients with the different Her2 scores considering different combinations of the scores (Figs. 3A-B and 4 A-B). This is maybe due to the limited number of positive cases and a shorter follow-up period of patients with positive cases since the patients have been followed for different lengths of time. Both patients with a score of 3 + were alive and doing well two years after surgery.

**Fig. 5:**
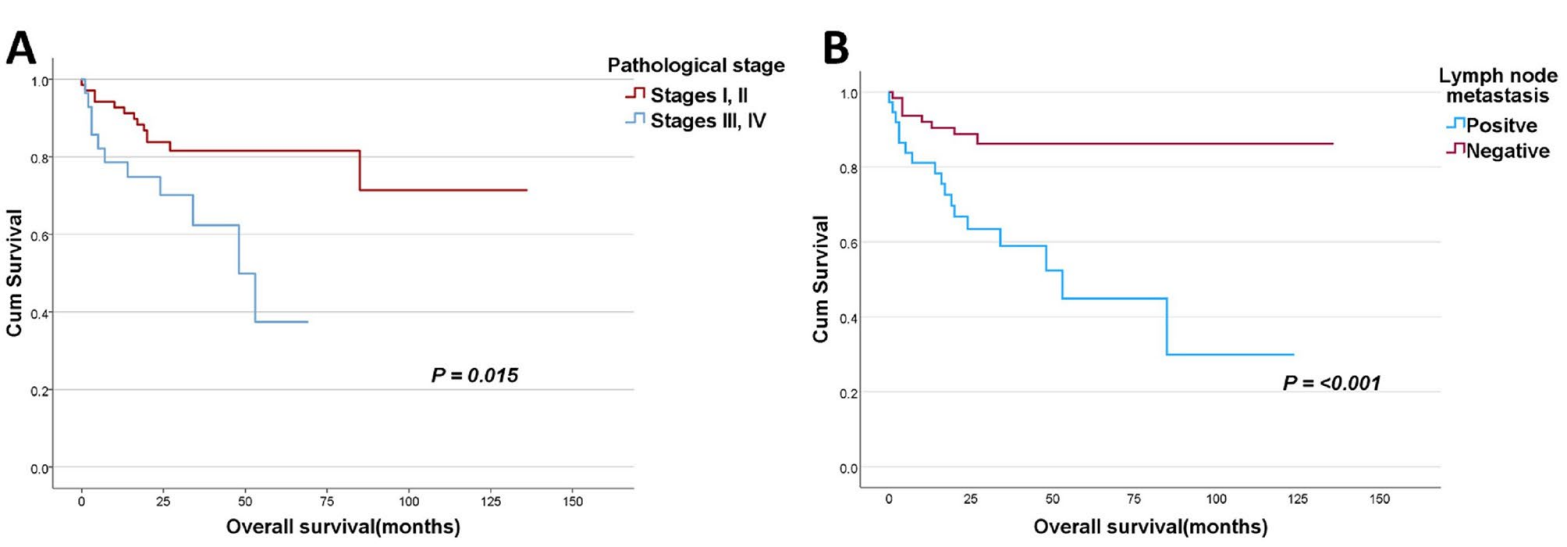
**Kaplan – Meier survival curves of overall survival according to the pathological tumor stage and lymph node metastasis** **A**: The correlation between the different pathological tumor stages and overall survival (OS). Tumor stages were divided into two groups, one composed of early stages I and II and another group comprised of late tumor stages III and IV; **B**: The correlation between lymph node metastasis status and overall survival (OS). OS was defined as the duration from the time of surgery to the time of death or the last follow-up appointment. P-values of ≤ 0.05 were regarded as statistically significant

Moreover, Analysis showed that pathological tumor stage and lymph node status significantly correlated with poor overall survival (advanced stages III and IV, *p* = 0.015, lymph node metastasis, *p* < 0.001, Fig. (5 A-B)); however, no significant association was found between survival and other described clinicopathological parameters.

## Discussion

Lung cancer is among the most prevalent cancers and the cause of worldwide cancer-related deaths. [34]. Despite the poor prognosis, the treatment outcomes for NSCLC have improved due to the discovery of frequent oncogenic drivers that guide treatment choice. Among these molecular changes, Her2 gene alterations are promising therapeutic targets. Several studies examined the effectiveness of Her2 targeted therapy in NSCLC; however, the results were mixed and differed according to the specific type of Her2 alterations.

In this study, we primarily aimed to assess the frequency of Her2 protein expression in NSCLC and its association with the clinicopathological features and prognosis among the Jordanian population. We observed a frequency of Her2 overexpression (score 3+) of 2% (2/100 cases) in NSCLC cases. One of the two positive cases was an adenocarcinoma (1/75, 1.3%), and the second was squamous cell carcinoma (1/24, 4.2%). This rate is comparable to some results reported in Asian populations [35–38] based on immunohistochemistry results, but it is lower than what was reported by other studies [17, 22, 26, 39, 40]. Variations in the reported frequencies may result from different reasons, including the proportion of histological subtypes, tissue processing and fixation conditions, the antibody used, and, most importantly, the lack of unified scoring criteria for interpreting the staining results. Most of the previous studies on Her2 expression in NSCLC defined the IHC positivity based only on the membranous staining, and many considered scores of 2 + and 3 + as positive, regardless of FISH results, so they reported a higher frequency of overexpression; however, when considering a score of 3 + as positive, they found a comparable rate to ours [17, 35]. On the other hand, Kim et al. used the H-scoring method that combines the cytoplasmic and membranous staining patterns and compared it to the membranous pattern only according to the ASCO/CAP guidelines and observed relatively comparable rates of overexpression (1.9% vs. 1.2% respectively) in lung adenocarcinoma cases [41]. Moreover, Miladinović et al. compared the results of Her2 expression in lung adenocarcinoma samples using two different antibodies, the HercepTest Dako and PATHWAY anti-HER2 (4B5) and they reported a lower rate of positivity using the 4B5 antibody (the one we used in this study), 2.7% vs. 6.4%. However, the overall agreement rate between these two antibodies was about 93.9% [42].

For the best interpretation of Her2 IHC results, the ASCO/CAP guidelines recommended specific tissue handling requirements, including putting the specimen in formalin as soon as it was resected, using the 10% neutral buffered formalin, and a fixation duration between 6 and 72 h [12]. Several studies investigated the effects of the preanalytical factors on the results of breast biomarkers, including delay to formalin fixation (DFF) (also called cold ischemia time), fixation type, and fixation duration. Lee et al. reported that a delay of formalin fixation of 1 and 8 h resulted in a reduced Her2 IHC expression in two tumors with a 3 + score using three different antibodies [43]. On the other hand, Moatamed et al. suggested that deviation from the recommended preanalytical factors of fixation type, fixation time (between 0 and 168 h), and a delay in fixation of 4 days at 4 °C may still show accurate results on HER2 IHC but in small core- sized specimens [44]. Further, Feng et al. suggested that using other fixative types than the recommended 10% NBF may have the potential to change the Her2 positive status to negative by IHC both in breast and gastric cancer tissues [45]. In this study, the specimens’ fixation conditions followed the recommended guidelines where the specimens were fixed in 10% NBF within 5 min of resection for a duration between 12 and 28 h. Therefore, the possibility that these preanalytical factors have affected the results of Her2 expression is relatively low.

In the current study, no statistically significant association was identified between Her2 expression and the different clinicopathological parameters, including age, gender, smoking, histological subtype, grade, stage, tumor size, lymph node status, and the predominant architectural pattern in ADC samples. This result is consistent with other results, which reported no association between Her2 overexpression and these clinicopathological features [22, 36, 37, 46, 47]. In contrast to our results, Suzuki et al. observed a significant correlation between Her2- positive cases by immunohistochemistry and lymph node metastasis and advanced tumor stages [37]. In addition, Cox et al. found a significant association between Her2 overexpression and advanced tumor stages [47]. Of note, Kim et al. found that Her2 overexpression is strongly associated with papillary predominant architectural pattern in adenocarcinoma cases [41]; our adenocarcinoma cohort does not include cases with papillary predominant architectural pattern to compare. Overall, in our case, due to the small sample size and the limited number of positive cases, we cannot draw a definite conclusion regarding these associations.

The incidence of Her2 gene mutation in NSCLC is between 2% and 4% [23, 37, 48], and it is more frequent in non-smoker females with Asian ethnicity and adenocarcinoma subtypes similar to those seen in EGFR (Her1) mutations. Her2 mutation consists of a heterogeneous group of alterations, and the most frequent mutation occurs in exon 20 [21, 37]. The results regarding the association between Her2 gene mutation and Her2 amplification and protein overexpression were conflicting. Suzuki et al. found that Her2 overexpression has a statistically significant association with Her2 mutation in NSCLC; however, immunohistochemistry showed low sensitivity and specificity for identifying the Her2 mutation. [37], and further, they found that Her2 amplification is present in about half of the cases with mutation. In contrast, Li et al. reported a negative association between Her2 amplification and mutation in lung cancer [23]. Further, the Her2 mutation’s prognostic value is unclear; several studies found no survival difference between those with or without the Her2 mutation [37, 49]. On the other hand, others have shown that Her2 mutation is associated with worse overall survival [38, 50]. In this study, in the very limited cohort of cases assessed for Her2 mutation, all cases were negative; and Her2 immunohistochemistry was not performed on these cases.

Unlike breast cancer and other malignancies, the prognostic significance of Her2 overexpression in NSCLC remains unclear. In this study, we did not find an effect of Her2 expression on OS or DFS, which probably can be explained by the limited number of positive cases and the short follow-up periods of these cases. Several previous studies also failed to find an association between Her2 overexpression and overall survival in NSCLC [17, 22, 37]. However, two large meta-analyses have shown that Her2 overexpression is an unfavorable prognostic factor in NSCLC, particularly in adenocarcinoma cases [20, 24]. More on survival analysis according to the other clinicopathological variables, our results demonstrated that advanced tumor pathological stages and the presence of positive lymph node metastasis significantly linked with bad overall survival in NSCLC (*p* = 0.015 and *p* < 0.001, respectively), which is consistent with previously reported results [51, 52]. On the other hand, no statistically significant association was identified between survival and the other clinicopathological features.

Although Her2 targeted therapy, in particular monoclonal antibodies alone or in combination with chemotherapy, was confirmed to have favorable effects on breast cancer treatment and had improving impacts on prognosis [13], the results of anti-Her2 therapy in NSCLC were conflicting. Many clinical studies tried to find the best treatment for Her2 alterations; however, their results were controversial. Some studies found no response to the Her2 monoclonal antibody Trastuzumab irrespective of the IHC or FISH status of Her2 [39, 53]. However, despite these discouraging results, few studies showed some promising results; of these, Gatzemeier et al., in a randomized phase II clinical trial, showed that in a very small subgroup of patients, composed of 6 patients harboring Her2+/FISH positive, the addition of trastuzumab to gemcitabine–cisplatin resulted in improved effects [25]. In another study conducted by de Langen et al. to assess the effect of trastuzumab and paclitaxel treatment in patients who were EGFR positive and had progression with the use of EGFR-TKI along with Her2 overexpression (IHC 1 + to 3 + or Her2 gene copy number > 1), they reported that about 46% out of the enrolled 24 patients showed an objective tumor response which was notably higher in those harboring Her2 score 3 + by IHC [54]. More interestingly, recently, the Food and Drug Administration (FDA) provided an accelerated approval to the use of the ADC trastuzumab deruxtecan (T-DXd) in patients with previously treated, unresectable or metastatic NSCLC carrying activating Her2 mutations. This was based on the efficacy analysis of DESTINY-Lung02 phase II clinical trial, which showed that patients treated with T-DXd at the 5.4 mg/kg dose level demonstrated a superior objective response rate with ORR of 57.7% [55]. However, despite these impressive results, the role of T-DXd in cases of NSCLC with Her2 overexpression needs more illustration. Further, Ado-trastuzumab emtansine (T-DM1), which is another ADC composed of trastuzumab linked to emtansine (an anti-microtubule agent) (DM1), has been proven to show a prominent efficacy in patients with Her2 mutation-positive NSCLC, particularly those with exon-20 insertion mutations [56]. These findings give hope toward successful Her2 targeted therapy and point to additional clinical studies with new strategies investigating the different Her2 alterations as distinct molecular targets.

There are some limitations to this study. First: this is a single institutional retrospective study, so some potential biases may exist. Further, this was reflected in the sample size, which was limited due to the lack of some patients’ clinical and follow-up information. Second, the patient’s follow-up periods had not been unified, affecting the validity of the survival analysis. Finally, and most importantly, we could not compare the results of the Her2 protein expression with those of the gene amplification or mutation.

## Conclusions

In summary, Her2 protein overexpression (score 3+) was detected in 2% of patients with NSCLC in a Jordanian cohort, which is somehow comparable to the rates observed in other Asian studies. No significant association was identified between Her2 expression and the variable clinicopathological parameters and survival. However, advanced pathological stages and positive lymph node metastasis were associated with an adverse overall survival. Although this study did not add significant findings and the number of positive cases was small, up to our knowledge; this is the first study to address the prevalence of Her2 protein expression in NSCLC among Middle East/ North Africa (MENA) region patients. Further, a high rate of Her2 low positive cases was observed, which may be considered for treatment assessment and may show some response to anti-Her2 drugs in a similar way to breast cancer. Since several clinical trials are testing different Her2-targeted therapies in NSCLC with different Her2 alterations, a large multi-institutional study is required to assess the Her2 abnormalities and their molecular associations since this may affect the outcome and the choice of treatment.

## Data Availability

The data used and analyzed during the current study are available from the corresponding author on reasonable request.

## List of Abbreviations

HER2: Human epidermal growth factor receptor 2
NSCLC: Non-small cell lung cancer
KHCC: King Hussein Cancer Center
IHC: Immunohistochemistry
ASCO/CAP: The American Society of Clinical Oncology/College of American Pathologists
EGFR: Epidermal growth factor receptor
ALK: Anaplastic lymphoma kinase
NGS: Next-generation sequencing
FISH: Fluorescence in situ hybridization
FFPE: Formalin-fixed, paraffin-embedded
H&E: Hematoxylin and eosin
ADC: Adenocarcinoma
SqCC: Squamous cell carcinoma
WHO: World Health Organization
AJC: American Joint Committee
NBF: Neutral buffered formalin
OS: Overall survival
DFS: Disease free survival
DFF: Delay to formalin fixation
